# The Psycho-Therapeutics of Insanity

**Published:** 1914-03-14

**Authors:** Bernard Hollander


### THE PSYCHO-THERAPEUTICS OF INSANITY.

By BERNARD HOLLANDER, M.D.

Anyone who has served in one of the Continental University Clinics for Mental Disorders, where daily and almost hourly fresh cases are brought in without any formality of certification, and patients are detained for a period of a few days or weeks until cured and discharged, or transferred to one of the State asylums if suffering from organic and more or less chronic disease, will confirm my observation that no institution for mental disorders is entitled to the designation of "hospital" unless its physicians are trained to apply those methods of psycho-therapeutics so essential to the recovery of a patient suffering from a psycho-neurosis, a recurring temporary insanity, or one of the typical insanities in the earliest stages. Psycho-therapy demands, of course, separate and individual treatment and consideration. We must study the temperament, character, and habits of each patient, as well as the circumstances of life which have brought out the mental disorder. No one has better reason to make a study of character than the physician in charge of the insane, and his conduct toward them will be modified, advantageously or otherwise, according to his skill in estimating human nature and individual peculiarities as they appear in persons suffering from disorders of the mind. If it is possible, as it generally is in the early stages of insanity, to treat the patient as a sick man, realising his own condition and wishing to co-operate in the measures taken for his relief, it is best to do so; the more nearly we can treat him as a rational being, the better, as a rule, we succeed with him.

### Suggestion Treatment.

It has been said that it is useless to attempt to convince a lunatic that his erroneous notions are not true. This is correct; discussion and argument are usually without avail. To contradict a patient would only irritate him, and to fall in with his delusion will only help to strengthen it. How are we, then, to correct his thoughts? First of all, by referring to his delusions as little as possible. We let him talk while we listen to him, but we take every opportunity to lead the conversation to another subject. The less a person indulges in his delusion the more it loses its force, until there comes a time and mood when skilfully selected arguments against his erroneous notions may be addressed to him, not obtrusively, but when occasion offers. Gradually the patient will suspect the falsity of his ideas, and may surprise one by saying: "You believe, then, I am suffering from a delusion?" A further sign of recovery is when the patient is able to explain how the erroneous and fixed idea arose, though his explanation is not always to be trusted.

Our aim is to produce rest to over-active parts of the brain. This is done either by directing the thoughts into other and healthy channels, as just explained, or, where this fails, to procure sleep for the patient. It is asserted that insane patients are not hypnotisable. This is true, because, as a rule, too much is attempted. Even with sane patients we do not nowadays attempt to produce the deep hypnotic stages. Since the insane we see in reception hospitals and observation clinics are usually not devoid of all reason, but have a good deal of logic and common sense preserved, which can be utilised by the operator, very fair success can be obtained among such patients if their consent and confidence can be gained. Deep sleep is unnecessary, nor need we make any suggestions; all we need try to accomplish is to attain a state of passivity in which the body and brain are resting, and the patient is in the greatest possible mental quietude. The melancholic requires peace, because painful impressions are excited by all psychic activity, even those that are in themselves normally pleasant; the maniac requires quietude because his cerebral excitement is otherwise intensified; exhausted patients require repose because every mental impression exhausts them still further. If, therefore, the patient can be taught to rest his brain we have gained a great deal. By making the patient listen attentively to a monotonous sound, or gaze intently on a particular object, we can exclude all disturbing and exciting thoughts and get him in a passive and subjective condition. Maybe the patient falls asleep; it is the best thing that can happen. Sleep often dissipates the fears and suspicions, allays the excitement and irritability of the patient, and brings him back his sane mind. But even without sleep it is evident that the patient who can rest quietly in the manner described has acquired a power, the exercise of which is most necessary to a sane mind —namely, the power of *self-control*. Gradually we may be able to introduce healthy mental complexes.

### Psycho-Analysis.

Many of the slighter mental troubles arise, especially in women with a neurotic taint, from the suppression of some emotional experience or of some desire condemned by her reason and conscience. Some mental or emotional shock has occurred in the past life of the patient and has become buried or forgotten in a state of sub-consciousness, where it becomes the cause of the patient's present mental symptoms; or some desire arising from a highly active instinct, which for conventional or other reasons had to be suppressed, leaves a trail in the brain-centres which shows itself in disordered function in later years. There is a lack of unity in the inner life because this suppressed thought or emotion or wish is not at one with the personality as a whole. By psycho-analysis the skilled psychologist lays bare the concealed cause of the patient's suffering, and thereby deprives the cause of its power to do harm.

This is, of course, a very brief and superficial account of what may be achieved by psycho-therapeutics in the treatment of mental disorders. It is the most effective weapon at our disposal, provided that any bodily ailment associated with the mental affection is duly attended to.

